# Endothelial Function as a Possible Significant Determinant of Cardiac Function during Exercise in Patients with Structural Heart Disease

**DOI:** 10.4061/2009/927385

**Published:** 2009-12-29

**Authors:** Bonpei Takase, Takashi Akima, Akimi Uehata, Masayuki Ishihara, Akira Kurita

**Affiliations:** ^1^Department of Intensive Care Medicine, National Defense Medical College, 3-2 Namiki, Tokorozawa, Saitama 359-8513, Japan; ^2^Saitama City Hospital, Saitama 359-8513 and Tokyo 154-0001, Japan; ^3^Self Defense Forces Central Hospital, Saitama 359-8513 and Tokyo 154-0001, Japan; ^4^Division of Biomedical Engineering, National Defense Medical College Research Institute, Saitama 359-8513 and Tokyo 154-0001, Japan; ^5^Fukuinkai Clinic, Saitama 359-8513 and Tokyo 154-0001, Japan

## Abstract

This study was investigated the role that endothelial function and systemic vascular resistance (SVR) play in determining cardiac function reserve during exercise by a new ambulatory radionuclide monitoring system (VEST) in patients with heart disease. The study population consisted of 32 patients. The patients had cardiopulmonary stress testing using the treadmill Ramp protocol and the VEST. The anaerobic threshold (AT) was autodetermined using the V-slope method. The SVR was calculated by determining the mean blood pressure/cardiac output. Flow-mediated vasodilation (FMD) was measured in the brachial artery to evaluate endotheilial function. FMD and the percent change f'rom rest to AT in SVR correlated with those from rest to AT in ejection fraction and peak ejection ratio by VEST, respectively. Our findings suggest that FMD in the brachial artery and the SVR determined by VEST in patients with heart disease can possibly reflect cardiac function reserve during aerobic exercise.

## 1. Introduction

Lipid abnormalities, hypertension, diabetes, smoking, and obesity are the traditional atherosclerotic risk factors. These risk factors cause endothelial dysfunction, inflammation, and altered biological pathways [1–3] which are well-recognized final common pathways that lead to the development of systemic artery atherosclerosis. These risk factors are also associated with impaired systemic circulation, which can also cause peripheral vascular remodeling. Peripheral vascular remodeling significantly influences systemic peripheral vascular resistance (SVR). SVR is thought to be one of the major determinants of cardiac function during exercise [1]. Thus, in patients with heart disease, peripheral vascular remodeling plays a key role in the pathophysiology of impaired exercise tolerance [4].

Flow-mediated vasodilation (FMD) in the brachial artery is one of the most widely used noninvasive diagnostic modalities [4, 5] for cardiovascular morbidity and mortality. However, though there are many studies dealing with endothelial function and SVR at rest, there is scant information about the relationship between peripheral vascular endothelial function and SVR during exercise. As well, the relationship between peripheral endothelial function and cardiac function reserve in response to exercise has not been fully investigated in patients with heart disease. This lack of information is related to the methodological difficulties that preclude precise evaluation of dynamic SVR and cardiac function during exercise [6].

However, recent advances in technology have enabled us to measure dynamic SVR and cardiac function during exercise at the same time. Recently, a new type of ambulatory radionuclide monitoring (VEST) system has been developed, which can be connected to computerized ambulatory-electrocardiogram monitoring using an IC card [7]. This VEST system is a useful and reliable piece of equipment for monitoring both SVR and left ventricular function during exercise [8, 9].

The purposes of the present study were (1) to investigate the role that peripheral endothelial function and dynamic changes in SVR during exercise have on left ventricular performance during exercise and (2) to clarify the relationship between peripheral endothelial function and SVR during exercise in patients with heart disease by using the new VEST system combined with cardiopulmonary function testing.

## 2. Methods

### 2.1. Study Population

The study population consisted of 32 men (63 ± 14 years old); 25 had coronary artery disease (including 14 with prior myocardial infarction), 3 had dilated cardiomyopathy, and 4 were diagnosed with hypertensive heart disease. Patients were excluded if they had acute myocardial infarction or decompensated heart failure within the last three months or severe valvular heart disease. All cardiac medications were discontinued for at least five half-lives before the study. All subjects were aware of the investigative nature of the study and gave their informed consent. The research protocol was approved by the Ethics Committee of our institute that follows the Declaration of Helsinki.

### 2.2. Equilibrium Radionuclide Ventriculography

All patients had ECG-gated blood-pool scintigraphy. After an injection of 740 MBq (20 mCi) of 99mTc-labeled human serum albumin into an antecubital vein, equilibrium radionuclide ventriculography was done at rest in the 45° left anterior oblique projection. The images were obtained using a gamma camera (GE Yokokawa, Hachiouji, Tokyo, Japan). The imaging data were taken in a 64 × 64 matrix with a 2.0 zoom, and were acquired for an average of 300 counts per pixel in the cardiac region of interest, with 24 frames per cardiac cycle using a computer system. The left ventricular ejection fraction was computed from the global time-activity curve using the single semiautomated method.

### 2.3. VEST System

To measure the serial changes in left ventricular function during cardiopulmonary stress testing, we used the newly developed VEST system (Capintec Inc., Ramsey, N.J.). This system consists of two radiation detectors and an ambulatory electrocardiographic recording system. The method employed when using this technique has been previously described [7]. Briefly, at the end of equilibrium radionuclide ventriculography, all patients wore the VEST garment and were positioned upright in front of the gamma camera. Then, one-radionuclide detector was positioned over the left ventricle while the other was placed over the right lung field attached to the VEST garment. The position of the detector was confirmed by acquiring a 30-second static image with the gamma camera.

After completion of cardiopulmonary stress testing, the patients were placed in front of the gamma camera and another 30-second static image was done to confirm that the VEST detector had not moved during the recording interval. Left ventricular function and the electrocardiogram were subsequently analyzed in a blind manner using the data recorded by the instrument.

### 2.4. Cardiopulmonary Stress Testing

After radionuclide ventriculography in the supine position, the patients assumed the upright position for symptom-limited cardiopulmonary stress testing using a Ramp treadmill protocol (Case 12, Marquette Electronics, Inc., Milwaukee, WI). Left ventricular function was assessed using the VEST system. Using a respiromonitor RM-300 and a gas analyzer MG-360 (AE280S, MINATO Ikagaku Inc., Osaka, Japan), breathing patterns were analyzed and oxygen uptake (VO_2_), carbon dioxide release, and ventilation were measured. Calibration of volumes and gases were carefully done prior to each test.

The 12-lead electrocardiogram was serially monitored, and the blood pressure was measured using the Korotkoff method every 60 seconds. The mean blood pressure was obtained using the following formula: mean blood pressure = diastolic blood pressure + (systolic blood pressure − diastolic blood pressure)/3. The double product was obtained by multiplying the systolic blood pressure by the heart rate. Before cardiopulmonary stress testing, baseline measurements were taken while the subjects stood for 5 minutes. Then, the patients underwent cardiopulmonary stress testing wearing the VEST system. After peak exercise, the recording was continued until the heart rate returned to the baseline rate. The end points for terminating exercise testing included: typical angina, dyspnea, extreme fatigue, or exercise-induced ST-segment depression >2 mm.

The anaerobic threshold (AT) was autodetermined using the V-slope method and a personal computer (NEC PC-9801) [10]. An analysis using the V-slope method was done using the gas exchange criteria at the point of nonlinear increase in the ventilatory equivalent for oxygen. This has been previously associated with elevations in simultaneously obtained arterial lactate samples without a simultaneous increase in the ventilatory carbon dioxide equivalent [11]. Heart rate, blood pressure, workload, VO_2_ at rest, AT, and peak exercise were recorded, and the measured VO_2_ was divided by the body weight.

### 2.5. Ultrasound FMD Measurements in the Brachial Artery

All ultrasound studies were done in a temperature-controlled room (25°C) with the subject in a fasting, resting, and supine state at approximately 08:00. Heavy meals, including a high-fat diet and caffeine-containing beverages, were prohibited from the night before the study. Blood pressure and heart rate were recorded from the left arm every 3 minutes with an automatic sphygmomanometer (Nihon Korin, BP-203, Tokyo, Japan) during the ultrasound procedure. A 7.5 MHz high-resolution ultrasound (Hewlett-Packard, SONOS 2000, Andover, MA, USA) was used to measure brachial artery diameter.

FMD was then measured by a previously validated technique [12]. Brachial artery diameter and flow velocity were imaged using a 7.5 MHz high-resolution ultrasound (Hewlett-Packard, SONOS 2000). For each subject, optimal brachial artery images were obtained between 2 and 10 cm above the antecubital fossa. After baseline measurements of brachial artery diameter and flow velocity, a small-width blood pressure cuff (Hokanson SC-10, Seattle, WA, USA) was inflated on the proximal portion of the forearm to occlusive pressure (200 mmHg) for 5 minutes in order to induce reactive hyperemia. The cuff was then rapidly deflated. Immediately after deflation, pulsed Doppler signals were recorded for 15 seconds. Two-dimensional images of the brachial artery were taken for 60 to 90 seconds after cuff deflation. The FMD was expressed as the percent change in diameter relative to the baseline diameter at rest. The average peak velocity was obtained from the pulsed Doppler signals recorded at rest and immediately after cuff deflation. The flow volume in the brachial artery was calculated by multiplying the average peak velocity and the vessel cross-sectional area ((*πD* ^2^)/4; *D* indicates the luminal diameter of the brachial artery) derived from the brachial artery diameter using the formula: brachial blood flow (ml/min) = average peak velocity (cm/s)/2 × *πD* ^2^ × 0.15. Reactive hyperemia was defined as the relative increase in the brachial blood flow calculated as the maximal flow measured immediately after cuff deflation divided by the flow obtained at rest (baseline). Finally, after a 15-minute interval, nitroglycerin-induced vasodilation (NTG-D) was performed. Baseline measurements of brachial artery diameter and flow velocity were again obtained, and 0.3 mg of sublingual nitroglycerin was then administered. Three minutes later, the brachial artery diameter was recorded. NTG-D was defined as the percent change relative to the baseline diameter. All images were recorded on super VHS videotape for later analysis. All ultrasound study and parameter measurements were performed by an investigator who was blinded to the other clinical information. A previous study in our laboratory showed that the intra- and interobserver variabilities (coefficient of variation) for repeated measures of diameter before and after reactive hyperemia in the brachial artery were <3% [12].

### 2.6. Data Analysis

With respect to the VEST recordings: The beat-to-beat data were taken over 30-second intervals, from which the end-diastolic volume, the end-systolic volume, the stroke volume, the left ventricular ejection fraction, the cardiac output, the left ventricular peak ejection ratio, and the left ventricular peak filling ratio were calculated. The background factor was corrected to obtain the best correlation with measurements taken with the gamma camera. The VEST system does not permit absolute measurements; end-diastolic volume data obtained at the beginning of the study were considered to be 100%, and subsequent measurements were expressed relative to this initial value. The SVR was calculated by dividing the mean blood pressure by the cardiac output obtained using the VEST measurements.

In order to assess the VEST parameters, changes from the values at 2 minutes after standing on the treadmill system (rest) to those at the AT point were calculated for the left ventricular ejection fraction, the left ventricular peak ejection ratio, and the SVR using the following formulas: changes in the left ventricular ejection fraction from rest to AT (%) = (left ventricular ejection fraction at AT − left ventricular ejection fraction at rest); changes in the left ventricular peak ejection ratio from rest to AT (%) = ((left ventricular peak ejection ratio at AT − left ventricular peak ejection ratio at rest) / left ventricular peak ejection ratio at rest × 100); changes in the SVR were also calculated using the same formulas as those for the left ventricular peak ejection ratio.

### 2.7. Statistical Analysis

Continuous variables are presented as the mean value ± standard deviation. Analysis of variance for repeated one-way ANOVA was applied to the data obtained at rest and during cardiopulmonary stress testing. When making multiple comparisons, significant probability values were defined and adjusted using the Bonferroni method. The relationships between variables were determined by doing simple linear regression analysis. A *P* value of less than 0.05 was considered statistically significant. All analyses were done on a personal computer with the statistical program Statview, Version 5.0 (SAS institute, Cary, NC).

## 3. Results

All patients exercised beyond AT (16.3 ± 3.3 mL/min/kg) until exhaustion without any ischemic ST-segment changes on electrocardiography. Heart rate and systolic blood pressure significantly increased from rest to AT or peak exercise. The double product and the body weight-corrected VO_2_ showed similar changes to the heart rate (Table 1).

The sequential data obtained using the VEST system are shown in Table 2. The left ventricular ejection fraction and stroke volume significantly increased from rest to AT or peak exercise. The left ventricular ejection fraction and left ventricular peak ejection ratio significantly increased from rest to AT or peak exercise (Table 2).

The FMD measurement results showed a significant increase of brachial artery diameter and calculated brachial artery blood flow (diameter, from 5.1 ± 0.7 mm to 5.2 ± 7.3 mm, *P* < .05; blood flow, from 15 ± 13 mL/sec to 65 ± 21 mL/sec, *P* < .05). From these data, the calculated FMD was 2.5% ± 2.0%, which was below the normal range obtained in our laboratory [13]. In contrast, NTG-D was 13.5 ± 4.3, which was within the normal limits obtained in our laboratory.

**Figure 1:**
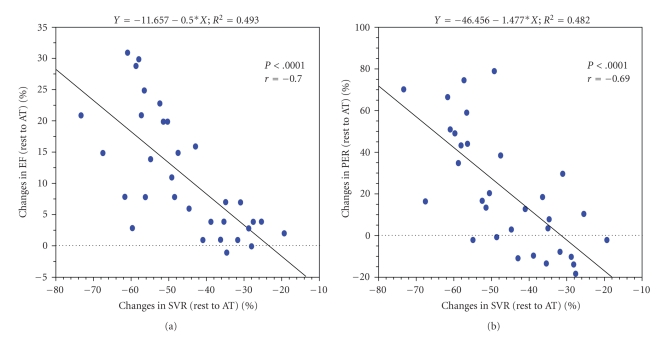
The correlation between the percent changes in systemic peripheral vascular resistance and cardiac response to exercise. EF: left ventricular ejection fraction; SVR: systemic peripheral vascular resistance; AT: anaerobic threshold; PER: peak ejection rate.

**Figure 2:**
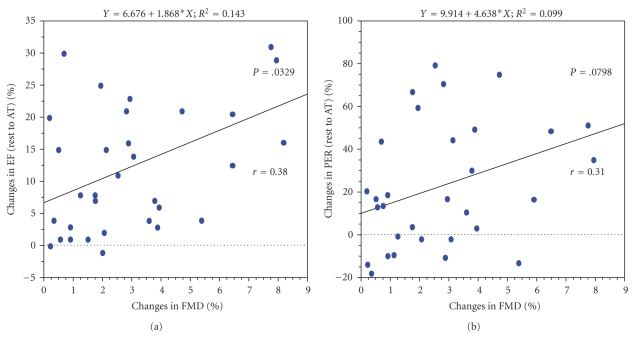
The correlation between flow-mediated vasodilation and cardiac response to exercise. FMD: flow-mediated vasodilation; EF: left ventricular ejection fraction; PER: peak ejection rate.

There was a significant inverse correlation between the changes in the SVR from rest to AT and the changes in left ventricular ejection fraction or left ventricular peak ejection ratio from rest to AT (Figure 1). FMD was significantly correlated with the change in left ventricular ejection fraction from rest to AT, and it tended to correlate with the changes seen in left ventricular peak ejection ratio from rest to AT (Figure 2). However, these relationships were not observed for NTG-D.

**Figure 3:**
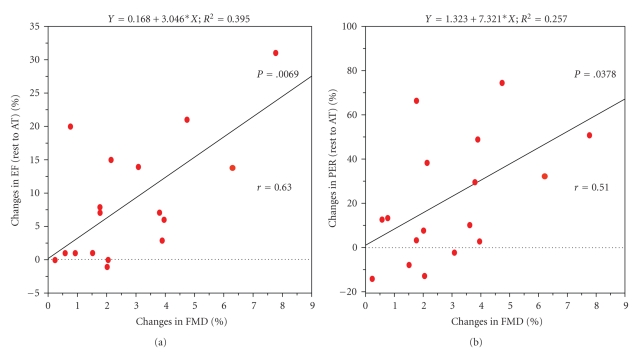
The correlation between flow-mediated vasodilation and cardiac response to exercise in the patients not on ACE-inhibitors. FMD: flow-mediated vasodilation; EF: left ventricular ejection fraction; PER: peak ejection rate.

**Figure 4:**
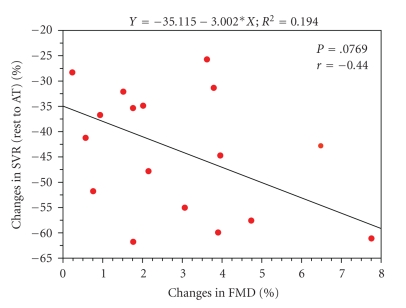
The correlation between flow-mediated vasodilation and changes in systemic peripheral vascular resistance during exercise in the patients not on ACE-inhibitors. FMD: flow-mediated vasodilation; SVR: systemic peripheral vascular resistance.

ACE-inhibitors have a potent effect on endothelial function (FMD); their use may confound the relationship between these various parameters [14, 15]. When FMD values were compared in patients with and without ACE-inhibitors, the patients with ACE-inhibitors tended to have better values of FMD than those without ACE-inhibitors (2.5 ± 1.9% versus 1.8 ± 2.2, *P* < .1). Thus, when patients on ACE-inhibitor treatment were excluded from the analysis, the FMD significantly correlated with both the left ventricular ejection fraction and the left ventricular peak ejection ratio change that occurred from rest to AT (Figure 3). In addition, if the correlation between the FMD and SVR during exercise was analyzed in this group of patients, there were a tendency to an inverse correlation (Figure 4).

## 4. Discussion

The present study has demonstrated that: (1) peripheral endothelial function possibly contributes to left ventricular performance during exercise; (2) SVR during exercise can determine left ventricular performance in response to aerobic exercise; and (3) peripheral endothelial function may partly contribute to SVR during aerobic exercise in patients with heart disease.

The correlation coefficient obtained in this study supports the possible presence of a significant relationship between peripheral endothelial function and left ventricular performance during aerobic exercise when patients on ACE-inhibitors were excluded. As well, a significant relationship between SVR and left ventricular performance was confirmed. In addition, peripheral endothelial function tended to correlate with the SVR obtained during aerobic exercise. Before reaching the anaerobic threshold, exercise is considered to be aerobic if no ischemia exists. In this study, no ischemic ECG changes were observed at the AT points in any of the patients studied. Thus, all of the patients were exercising aerobically when the contributions of peripheral endothelial function and SVR on left ventricular performance were observed. However, when the relatively low correlation coefficient *r* value and the influence of ACE-inhibitors were considered, the definite role of peripheral endothelial function on left ventricular performance during exercise should be confirmed by duplicated larger cohort study of our observation.

By using a combination of the VEST system and blood pressure measurements [16], we could calculate dynamic SVR. The changes in the SVR that occur during exercise reflect peripheral vasomotion during exercise. The VEST system is useful for evaluating not only serial cardiac function but also peripheral vasomotion during exercise in the upright position. By using this device, one can simultaneously evaluate left ventricular ejection fraction, cardiac output, stroke volume, left ventricular end diastolic volume, left ventricular end systolic volume, and the left ventricular peak ejection ratio [17, 18].

The findings in this study give a new insight into the exercise physiology of patients with heart disease. Cardiac performance is closely associated with dynamic SVR during exercise obtained using the new VEST system. Cardiopulmonary stress testing in combination with the VEST system can be more widely applied in clinical practice to help manage heart disease. Using this modality, we can evaluate the efficacy of a new medication or a new therapeutic option, such as biventricular pacing, in patients with heart failure. Furthermore, the mechanisms of action of new treatments can also be elucidated.

Peripheral endothelial function contributes not only to resting cardiac function but also to the dynamic cardiac function change that occurs during exercise. One could speculate that direct and indirect mechanisms are responsible for this phenomenon. However, the present study's findings cannot help clarify these mechanisms. While in one study nitric oxide aggravated cardiac function in patients with heart disease [19], other reports have shown that nitric oxide improved cardiac function in the failing heart [20, 21]. There are three distinct nitric oxide synthases (NOS), neural (nNOS), inducible (iNOS), and endothelial (eNOS), that probably behave differently in the failing heart at rest and during exercise. Generally, nitric oxide is reported to modulate *β* adrenergic responsiveness [22], and nitric oxide can regulate myocardial oxygen consumption (MVO_2_) in the failing heart [23, 24]. Therefore, nitric oxide could possibly affect cardiac performance even during exercise [25]. With respect to indirect mechanisms, exercise-induced hyperemia in either skeletal or cardiac muscle is at least partly nitric oxide-dependent [26]. A previous report found that nitric oxide was related to microvascular function in humans [27]. Exercise training and exercise therapy in patients with heart disease improved both endothelial function and peripheral vascular circulation [28–32]. Brachial artery endothelial function may be indirectly associated with cardiac function during exercise. Since endothelial function could possibly be related with cardiac function during exercise, medical therapy, including prescribing ACE-inhibitors, statins and/or antioxidant therapy, as well as exercise training, might improve cardiac function during exercise by improving endothelial function in patients with heart disease.

In our study, brachial (peripheral) endothelial function tended to correlate with the systemic vascular response that occurred during exercise. However, this tendency was only observed when patients on ACE-inhibitors were excluded from the analysis, as ACE-inhibitors have potent effects on endothelial function even after having been discontinued for 24 hours [14, 15]. There are many neurohormonal factors that play a role in exercise physiology and pathophysiology [33]. Among them, nitric oxide has been reported to be involved in 20%–40% of the entire control of the vasomotion during exercise. Thus, the moderate correlation obtained in this study agrees with previously reported findings.

Study limitations: this study has several limitations. First, the study population was not homogeneous; thus, different baseline characteristics might have confounded the main findings of the present study. This study should be duplicated with more homogeneous populations. Second, endothelial function during exercise could not be measured due to technical problems. If a new technology that measures endothelial function during exercise could be developed, the role of endothelial function in systemic vascular resistance and left ventricular function during exercise would be further elucidated. Third, since recently statisticians criticize investigators for utilizing the correlation analysis between the changes in two measured values [34] and recommend to use Blomquist's formula, our results might have less impact on the relation between peripheral endothelial function and left ventricular performance during exercise. However, the criticizing report is relatively new and, in addition, commercially available personal computer package of this statistics does not exist so that we could not analyze our data by using Blomquist's formula. Our findings should be further confirmed by new analysis in the near future. Fourth, by the recent advance of technology, FMD and NTG-D now can be measured by on-line system [35] so that our method of measuring FMD and NTG-D is somewhat out of date. We did not have duplex measurements to obtain flow and shear rates, and the time to peak for FMD could not be determined by our method. In contrast, recent report [35] especially showed that the time to peak for FMD exceeded more than 90 seconds. In addition, about NTG-D, on-line continuous measurement for NTG-D, is supposed to be important to obtain peak NTG-D value. These somewhat out-of-date methods likely confound the interpretation of FMD, NTG-D and the reactive hyperemic response of brachial artery flow. Our data again should be duplicated in recent sophisticated equipment. However, since the huge amount of articles on FMD and NTG-D have been published by using same measurement method as ours, our findings have significant message on the relation between peripheral endothelial function and left ventricular performance during exercise. Lastly, our Vest system can only measure relative change of left ventricular dimension so that absolute value of the left ventricular volume parameters such as stroke volume that directly influence exercise tolerance could not be evaluated. This is another limitation of this study.

Despite these limitations, we can conclude that peripheral endothelial function and vasomotion, including SVR, might play a significant role in determining cardiac performance during exercise in patients with heart disease. This information provides a new insight into the understanding of the exercise physiology of patients with heart disease.

## Figures and Tables

**Table 1:** The summary of cardiopulmonary stress testing.

	Rest	AT	Peak	*P*-values
				Rest versus AT or Peak
HR (bpm)	72 ± 13	101 ± 12	130 ± 14	<.01
SBP (mmHg)	120 ± 16	133 ± 23	156 ± 22	<.01
DBP (mmHg)	68 ± 10	71 ± 9	74 ± 10	.29
VO_2_/W(mL ∗ min^−1^ ∗ kg^−1^)	4.5 ± 2.1	16.3 ± 3.3	24.2 ± 7.5	<.01

Data are presented as the mean value ± standard deviation. HR: heart rate; SBP: systolic blood pressure; DBP: diastolic blood pressure; VO_2_/W: oxygen uptake corrected by body weight; AT: anaerobic threshold point; Peak: at peak exercise.

**Table 2:** The summary of the selected parameters obtained from VEST system.

	Rest	AT	Peak	*P*-values
				Rest versus AT or Peak
EF (%)	54 ± 12	65 ± 16	57 ± 10	<.01
PER (EDV0∗sec^−1^)	3.1 ± 0.9	3.6 ± 1.0	4.3 ± 0.9	<.01
SVR (mmHg ∗ EDV0^−1^ ∗ min^−1^)	2.6 ± 0.8	1.3 ± 0.4	1.2 ± 0.2	<.01

Data are presented as the mean value ± standard deviation. EF: ejection fraction; PER: peak ejection ratio; SVR: systemic vascular resistance; AT: anaerobic threshold; Peak: at peak exercise.

## References

[1] Braunwald E (1977). Vasodilator therapy—a physiologic approach to the treatment of heart failure. *The New England Journal of Medicine*.

[2] Schächinger V, Britten MB, Zeiher AM (2000). Prognostic impact of coronary vasodilator dysfunction on adverse long-term outcome of coronary heart disease. *Circulation*.

[3] Anderson TJ, Gerhard MD, Meredith IT (1995). Systemic nature of endothelial dysfunction in atherosclerosis. *The American Journal of Cardiology*.

[4] Nakamura M (1999). Peripheral vascular remodeling in chronic heart failure: clinical relevance and new conceptualization of its mechanisms. *Journal of Cardiac Failure*.

[5] Celermajer DS, Sorensen KE, Gooch VM (1992). Non-invasive detection of endothelial dysfunction in children and adults at risk of atherosclerosis. *The Lancet*.

[6] Sarnoff SJ, Berglund E (1954). Ventricular function. I. Starling's law of the heart studied by means of simultaneous right and left ventricular function curves in the dog. *Circulation*.

[7] Hosaka H, Takase B, Kitamura K (2001). Assessment of left ventricular volume by an ambulatory radionuclide monitoring system during head-up tilt in patients with unexplained syncope: relation to autonomic activity assessed by heart rate variability. *Journal of Nuclear Cardiology*.

[8] Tamaki N, Strauss HW (1987). Assessment of cardiac function by an ambulatory ventricular function monitor (VEST)—(2). Application in coronary artery disease. *Kaku Igaku*.

[9] Nappi A, Cuocolo A, Imbriaco M (1997). Ambulatory monitoring of left ventricular function: walk and bicycle exercise in congestive heart failure. *The Journal of Nuclear Medicine*.

[10] Anderson SJ, Hughson RL, Sherrill DL, Swanson GD (1986). Determination of the “anaerobic threshold”. *Journal of Applied Physiology*.

[11] Blachura L, Emmerich J, Stoklosa J, Plucinska G (1984). The level of anaerobic threshold determined from the gas exchange and acid-base equilibrium of the blood in women and men. *Acta Physiologica Polonica*.

[12] Uehata A, Lieberman EH, Gerhard MD (1997). Noninvasive assessment of endothelium-dependent flow-mediated dilation of the brachial artery. *Vascular Medicine*.

[13] Etsuda H, Takase B, Uehata A (1999). Morning attenuation of endothelium-dependent, flow-mediated dilation in healthy young men: possible connection to morning peak of cardiac events?. *Clinical Cardiology*.

[14] Mancini GBJ, Henry GC, Macaya C (1996). Angiotensin-converting enzyme inhibition with quinapril improves endothelial vasomotor dysfunction in patients with coronary artery disease: the TREND (Trial on Reversing ENdothelial Dysfunction) Study. *Circulation*.

[15] Anderson TJ, Elstein E, Haber H, Charbonneau F (2000). Comparative study of ACE-inhibition, angiotensin II antagonism, and calcium channel blockade on flow-mediated vasodilation in patients with coronary disease (BANFF study). *Journal of the American College of Cardiology*.

[16] Ciampi Q, Betocchi S, Violante A (2003). Hemodynamic effects of isometric exercise in hypertrophic cardiomyopathy: comparison with normal subjects. *Journal of Nuclear Cardiology*.

[17] Cuocolo A, Storto G, Izzo R (1999). Effects of valsartan on left ventricular diastolic function in patients with mild or moderate essential hypertension: comparison with enalapril. *Journal of Hypertension*.

[18] Todino V, Rubini G, Cuocolo A (1999). Assessment of left ventricular function by ECG-gated myocardial perfusion scintigraphy with image inversion technique: comparison with equilibrium radionuclide angiography. *Journal of Nuclear Cardiology*.

[19] Sevransky J, Vandivier RW, Gerstenberger E (2005). Prophylactic high-dose N*ω*-monomethyl-L-arginine prevents the late cardiac dysfunction associated with lethal tumor necrosis factor-*α* challenge in dogs. *Shock*.

[20] Bendall JK, Damy T, Ratajczak P (2004). Role of myocardial neuronal nitric oxide synthase-derived nitric oxide in *β*-adrenergic hyporesponsiveness after myocardial infarction-induced heart failure in rat. *Circulation*.

[21] Setty S, Tune JD, Downey HF (2004). Nitric oxide contributes to oxygen demand-supply balance in hypoperfused right ventricle. *Cardiovascular Research*.

[22] Shinke T, Takaoka H, Takeuchi M (2000). Nitric oxide spares myocardial oxygen consumption through attenuation of contractile response to beta-adrenergic stimulation in patients with idiopathic dilated cardiomyopathy. *Circulation*.

[23] Traverse JH, Chen Y, Hou M, Bache RJ (2002). Inhibition of NO production increases myocardial blood flow and oxygen consumption in congestive heart failure. *American Journal of Physiology, Heart and Circulatory Physiology*.

[24] Chen Y, Traverse JH, Du R, Hou M, Bache RJ (2002). Nitric oxide modulates myocardial oxygen consumption in the failing heart. *Circulation*.

[25] Parent R, Leblanc N, Lavallee M (2006). Nitroglycerin reduces myocardial oxygen consumption during exercise in spite of vascular tolerance. *American Journal of Physiology, Heart and Circulatory Physiology*.

[26] Green DJ, Maiorana A, O'Driscoll G, Taylor R (2004). Effect of exercise training on endothelium-derived nitric oxide function in humans. *Journal of Physiology*.

[27] Wang J-S (2005). Effects of exercise training and detraining on cutaneous microvascular function in man: the regulatory role of endothelium-dependent dilation in skin vasculature. *European Journal of Applied Physiology*.

[28] Borland C (2004). Nitric oxide diffusing capacity on exercise. *Chest*.

[29] Maeda S, Tanabe T, Otsuki T (2004). Moderate regular exercise increases basal production of nitric oxide in elderly women. *Hypertension Research*.

[30] Steiner S, Niessner A, Ziegler S (2005). Endurance training increases the number of endothelial progenitor cells in patients with cardiovascular risk and coronary artery disease. *Atherosclerosis*.

[31] Sackner MA, Gummels E, Adams JA (2005). Effect of moderate-intensity exercise, whole-body periodic acceleration, and passive cycling on nitric oxide release into circulation. *Chest*.

[32] Banfi G, Malavazos A, Iorio E (2006). Plasma oxidative stress biomarkers, nitric oxide and heat shock protein 70 in trained elite soccer players. *European Journal of Applied Physiology*.

[33] Maxwell AJ, Schauble E, Bernstein D, Cooke JP (1998). Limb blood flow during exercise is dependent on nitric oxide. *Circulation*.

[34] Yu Y, Glithorpe MS (2007). Revising the relation between change and initial value: a review and evaluation. *Statistics in Medicine*.

[35] Black MA, Cable NT, Thijssen DHJ, Green DJ (2008). Importance of measuring the time course of flow-mediated dilatation in humans. *Hypertension*.

